# Littre’s hernia—a reason for resection

**DOI:** 10.1093/jscr/rjac617

**Published:** 2023-01-10

**Authors:** David Seok, Silvy Akrawe, Vijay Mittal

**Affiliations:** Department of General Surgery, Ascension Providence Hospital and Medical Center, Southfield, MI, USA; Department of General Surgery, Ascension Providence Hospital and Medical Center, Southfield, MI, USA; Department of General Surgery, Ascension Providence Hospital and Medical Center, Southfield, MI, USA

## Abstract

Littre’s hernias are exceedingly rare, with an estimated incidence of 0.09% in strangulated or incarcerated hernias. It may present as a complication of Meckel’s diverticulum in less than 1%. In the adult population, it presents as an inguinal (50%), femoral (20%) and umbilical (20%) hernias. Management of Meckel’s diverticulum when encountered during a routine repair of hernia in literature is generally resection. We present a case of a healthy 68-year-old gentleman who was found to have a Meckel’s diverticulum in the indirect hernia sac during routine elective open right inguinal hernia repair.

## INTRODUCTION

Littre’s hernia (LH) is any hernia with the sac containing a Meckel’s diverticulum (MD) and is named after the French surgeon Alexis de Littré who reported the case first in the 1700s. LH is exceedingly rare with an estimated incidence of 0.09% of strangulated or incarcerated hernias in literature [1] and can be seen as a complication of MD in less than 1% of cases.

LH can occur in both the adult and pediatric population. In children, it is thought to be more common to have them occur in umbilical hernias [2]. In the adult population, it may present as an inguinal—usually right-sided (50%), femoral (20%) or umbilical hernia (20%). And although uncommon, it can occur as other hernias [1–3]. Given the rare incidence of an LH, it has been suggested that every case of how it presents and is managed should be documented, as about only 50 cases have been documented in literature [4].

When an MD is encountered incidentally during operation, resection is generally recommended for all symptomatic MD. Classic symptoms associated with MD are bleeding, ulceration, perforation and sometimes strangulation. Simple transverse diverticulectomy is preferred and may suffice. However, if the MD has a broad base, a small palpable mass or an ulcer, then a wedge resection may be a better alternative [5, 6]. Segmental small bowel resection and anastomosis may be required.

Management of asymptomatic and incidentally found MD is less clear. Prophylactic resection may be pursued based on various risk factors, such as male sex, age younger than 45 years, presence of a fibrous band or long diverticula [5]. Management of MD when encountered during a routine repair of hernia in literature is generally resection [7], though the reasoning behind this is not elucidated.

## CASE PRESENTATION

We present a case of a relatively healthy 68-year-old gentleman who presented for open right inguinal hernia repair. He had first noticed a bulge in the right groin about 2 years ago. He noted some soreness after coughing and strenuous exercise. On physical exam, a defect in the inguinal region was appreciated, without the presence of bowel. Medical history included a diagnosis of type 2 diabetes mellitus without the need of exogenous insulin and atrial fibrillation for which ablative therapy was performed.

**Figure 1:**
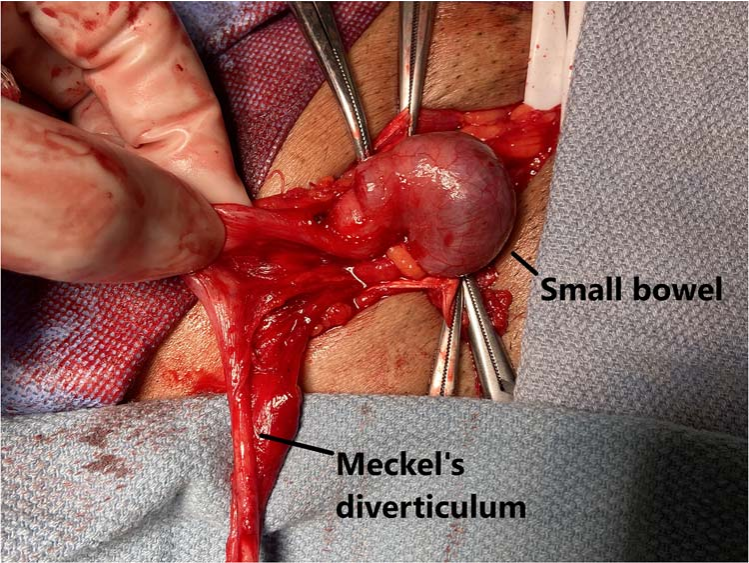
Meckel’s diverticulum identified after opening of the hernia sac.

**Figure 2:**
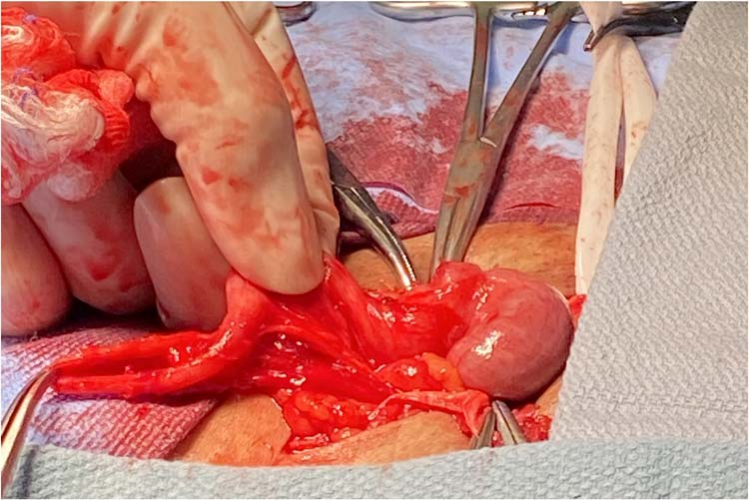
Meckel’s diverticulum attached to small bowel.

An open right inguinal hernia repair using Lichtenstein tension-free mesh repair was performed. A 6-cm oblique skin incision was made, the external oblique aponeurosis was opened and the cord structures were isolated. An indirect hernia was identified, and within the sac, a cord-like structure similar in feeling to that of the vas deferens was noted. The sac was opened and freed from the cord-like structure, which was adherent to the sac. It appeared to be a long-tubular structure similar in appearance to the appendix, but without a mesentery (Fig. 1). When traced proximally, it appeared to be originating from the antimesenteric side of a loop of small bowel, making it a Meckel’s diverticulum (Fig. 2). It measured 8.5 × 0.7 × 0.6 cm. The MD was resected at the base using a surgical stapler (EndoGIA™, Medtronic, MN) and sent for pathology. The remainder of the tension-free mesh repair was completed using polypropylene mesh (ProGrip™, Medtronic, MN).

The patient was discharged home with medications for pain control, instructions on wound care and notified of the interesting finding. Pathological examination found no masses or lesions inside the lumen of the diverticulum. Intestinal mucosa was seen, but no other pathology was encountered. The patient was seen 1 week post-operatively in the clinic and has progressed without further issues.

## DISCUSSION

When encountered in an asymptomatic setting during routine hernia repair, the finding of a MD in the hernia sac can be surprising. The management in literature favors resection [4, 7, 8]. We agree with this approach, when MD is found in the hernia. This is on the basis that the hernia may possibly be a symptom of MD. Whether a diverticulectomy, wedge resection or segmental intestinal resection should be performed should be dictated based on surgical principles that relate to MD. If the MD has a broad base, a small palpable mass or signs of chronic ulceration, a wedge or segmental resection may be warranted. Of course, with the use of permanent mesh, the risks of mesh infection are increased. As such, it is imperative that sterile surgical technique is maintained throughout the case, and especially during resection.

The exact pathologic process of LH is unknown. Being a rare entity in and of itself, having a MD in a hernia sac may be assumed as a random occurrence. One theory may be that an inflammatory inciting event caused the MD to be adherent to the peritoneum prior to the descent of the testes. Then, during the development of the processus vaginalis, the MD that was adherent to the peritoneum was carried down. The presence of it may not allow for the closure of the processus vaginalis, thereby keeping it patent. Perhaps a similar situation occurs in umbilical hernias in the pediatric population.

In regards to the histopathologic analysis of the resected specimen, ours was of only intestinal tissue. It is not unusual to find ectopic tissue in MD such as gastric (23–50%) and pancreatic (5–16%) [7].

## CONCLUSION

MD found in an inguinal hernia should be managed as a symptomatic MD and be resected.
